# Influence of Environmental Exposures on the Mechanical Performance and Durability of 3D-Printed Cementitious and Alkali-Activated Composites

**DOI:** 10.3390/ma19153185

**Published:** 2026-07-25

**Authors:** Magdalena Rudziewicz, Marcin Maroszek, Karina Rusin-Żurek, Marek Hebda

**Affiliations:** 1 Faculty of Materials Engineering and Physics, Cracow University of Technology, Warszawska 24, 31-155 Kraków, Poland; magdalena.rudziewicz@doktorant.pk.edu.pl (M.R.); marcin.maroszek@doktorant.pk.edu.pl (M.M.); karina.rusin-zurek@pk.edu.pl (K.R.-Ż.); 2 Faculty of Mechanical Engineering, Cracow University of Technology, Warszawska 24, 31-155 Kraków, Poland

**Keywords:** additive manufacturing, alkali-activated binders, environmental exposure, 3D-printed concrete, foamed materials, frost resistance, merino wool fibres

## Abstract

This study evaluates the mechanical performance, anisotropy, and spatial variability of 3D-printed cementitious and alkali-activated composites under laboratory, atmospheric, and freeze–thaw conditions. Alkali-activated composites exhibited substantially higher shrinkage than cement-based mixtures, reflecting differences in their reaction mechanisms and pore structure development. Compressive strength was measured in two orthogonal directions representing perpendicular (⟂) and parallel (∥) behaviour. Non-activated mixtures exhibited compressive strength of 11–12 MPa, whereas alkali-activated composites reached 19–20 MPa in the reference condition. Atmospheric exposure increased compressive strength by 10–22%, while freeze–thaw cycles did not significantly affect perpendicular strength. Flexural strength of non-activated mixtures remained low (3.7–5.3 MPa), whereas activated composites showed higher values in the reference state (8.8–15.0 MPa) but decreased after atmospheric exposure to 5.3–5.8 MPa. The degree of anisotropy increased significantly for alkali-activated mixtures (from 0.09 to 0.24) while remaining relatively stable for non-activated materials (0.04–0.11). Glass fibres showed no significant degradation after environmental and freeze–thaw exposure, while merino wool fibres exhibited only minor surface irregularities, confirming the potential of both fibre types for use in sustainable lightweight 3D-printed cementitious and alkali-activated composites.

## 1. Introduction

The construction sector is currently facing the challenge of simultaneously reducing greenhouse gas emissions, limiting the consumption of primary raw materials, and improving the energy efficiency of buildings [1,2,3,4]. In addition to the decarbonisation of cement production, increasing importance is being given to the development of materials with reduced bulk density and enhanced thermal insulation performance, which may contribute to lowering energy demand during the operational phase of buildings [5,6,7]. In this context, particular attention is paid to porous cement-based composites [8] and foamed concretes [8], owing to their reduced density and potential for developing lightweight materials suitable for extrusion-based 3D printing [9,10]. Materials characterised by increased porosity, however, exhibit reduced mechanical parameters and greater sensitivity to environmental factors such as moisture or cyclic freezing and thawing [11,12]. Therefore, an important research direction is their modification through the incorporation of dispersed fibre reinforcement, which can limit the development of microcracks, improve structural integrity, and stabilise material properties over time [13]. Additive manufacturing of cementitious materials has recently gained significant attention as a promising technology enabling automated construction and optimised material usage. In the case of extrusion-based 3D-printed cementitious materials, fibres may perform an additional function by enhancing interlayer bonding and improving load transfer between layers, which can contribute to reducing the anisotropic mechanical behaviour resulting from the layer-by-layer deposition process [14,15].

**Figure 1:**
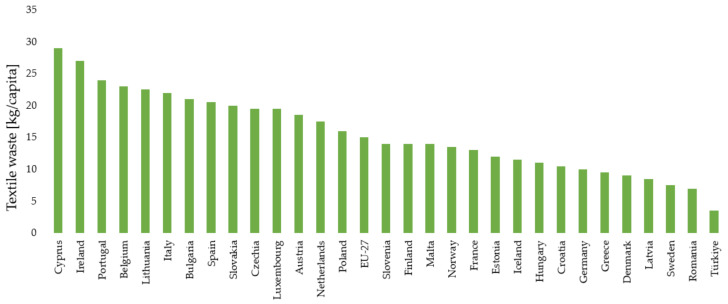
The distribution of textile waste generation in 2020, in kg per capita [21].

At the same time, the importance of the circular economy (CE) is increasing, in which post-production waste is treated as a valuable secondary raw material [16,17]. One significant yet still insufficiently utilised waste stream consists of textile fibre waste, including wool waste generated by the clothing and textile processing industries [18,19,20]. In 2020, the EU-27 generated approximately 6.95 million tonnes of textile waste, corresponding to about 16 kg per capita. Of this amount, only around 4.4 kg per person was collected separately for reuse and recycling, while the remaining 11.6 kg per capita was disposed of together with mixed municipal waste. This indicates that a substantial share of textile waste remains insufficiently recovered and continues to be directed to conventional waste streams. Figure 1 presents the distribution of textile waste generation by country, expressed in kilograms per capita [21]. Natural fibres, such as wool, are characterised by low density, a developed surface structure, and the ability to absorb moisture, which may influence the microstructure of porous composites and their thermal insulation properties [22,23]. At the same time, their hygroscopic nature and susceptibility to degradation in the alkaline environment of the cementitious matrix constitute significant limitations to durability [24]. In this context, the incorporation of textile-derived fibres into lightweight, porous cementitious composites represents a promising strategy for both waste valorisation and functional material development. However, achieving a balance between improved microstructural performance, thermal insulation potential, and long-term durability remains a significant challenge, particularly in materials manufactured using extrusion-based 3D printing.

Glass fibres, commonly used in cement composites, exhibit high tensile strength, dimensional stability, and greater chemical resistance [25]. However, their production is associated with significant energy consumption, and the waste generated during industrial processes and the manufacturing of glass fibre-reinforced polymer composites requires appropriate management and valorisation [26,27,28]. The incorporation of glass fibres originating from production waste into lightweight cement composites may therefore constitute an element of a strategy aimed at reducing the environmental impact of the construction sector. An increasingly proposed solution is hybrid reinforcement, which involves combining natural and technical fibres within a single matrix [29]. Studies indicate that combining fibres with different stiffness and chemical characteristics may lead to a synergistic effect, in which natural fibres limit microcrack initiation and modify the pore structure, while technical fibres stabilise the mechanical response of the composite under higher loads [30,31]. In the case of highly porous materials, understanding the influence of fibres on pore size distribution and morphology, moisture transport, and the behaviour of the material under variable temperature conditions is particularly important.

Although considerable research has been conducted on the use of natural and technical fibres in cementitious composites, limited knowledge is still available regarding their combined performance in lightweight, porous materials produced by 3D printing, particularly in terms of long-term durability and resistance to environmental exposure. In highly porous structures, moisture transport and repeated freeze–thaw cycles may accelerate the degradation of natural fibres and reduce the bond strength at the fibre–matrix interface [32,33]. At the same time, porosity plays a crucial role in determining the thermal insulation performance of cementitious materials, requiring a balance between density, mechanical strength, and thermal conductivity. Therefore, the aim of this study is to evaluate the influence of hybrid fibre reinforcement, combining waste textile fibres (merino wool) with glass fibres in various proportions, on the fresh, mechanical, and durability properties of lightweight, porous cementitious composites produced using 3D-printing technology. The analysis includes processing-related parameters (mixture preparation and print stability), shrinkage, resistance to freeze–thaw cycles, and changes in strength and interfacial bonding after laboratory and atmospheric exposure (after six months), as well as the microstructure and the degree of fibre degradation.

## 2. Materials and Methods

### 2.1. Materials

**Figure 2:**
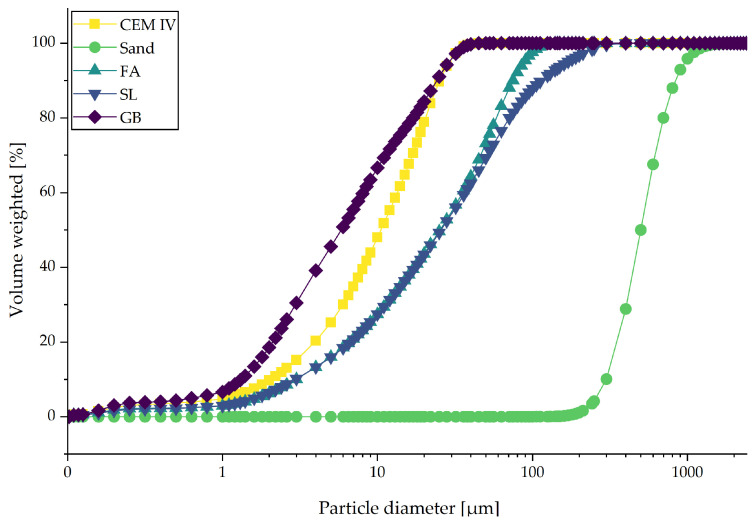
Cumulative particle size distribution (volume weighted).

The reference mixture composition was developed in the authors’ previous research on 3D printing of foamed concrete (3DFCP) and was based on CEM IV/B(V) 42.5 N cement. The primary raw materials used included quartz sand and low-emission ordinary Portland cement (OPC), while the recycled components comprised ground demolition brick (GB), fly ash (FA), and coal slag (SL) [34]. It should be emphasised that the selection of mixtures for the present stage of research was performed in a screening manner, based on the outcomes of the authors’ earlier experimental investigations [35,36,37]. In the current study, the selected formulations were further modified and optimised while employing the same base materials, the characteristics of which are presented in Figure 2 and Table 1 and Table 2. To enhance the structural performance of the mixtures, glass fibres (GFs) and merino wool fibres (MFs), both sourced from post-industrial waste, were used as reinforcement. The water-to-cement ratio (*w*/*c*) was calculated as the ratio of the total mass of all liquid constituents to the total mass of all dry powder constituents used in the mixture.

**Figure 3:**
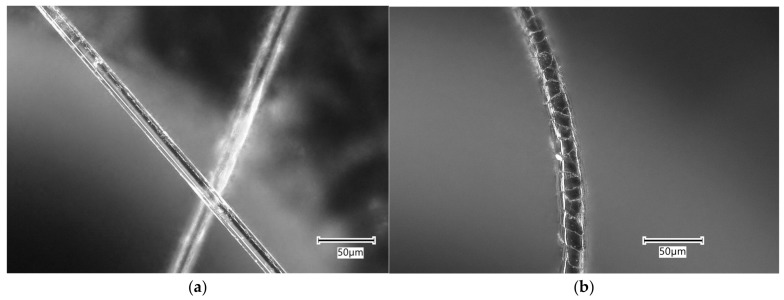
Surface of glass fibres (**a**) and merino wool fibres (**b**).

Figure 3 shows the morphology of merino wool fibres, revealing a characteristic scaly surface structure resulting from the keratinous composition of the fibre. Wool fibres exhibited a rough and irregular surface with visible cuticle scales, which may enhance mechanical interlocking with the surrounding matrix. In contrast, glass fibres exhibited a relatively smooth and homogeneous surface, which contributes to their high mechanical strength and stable bonding behaviour within cement-based composites [46].

The complete mixture compositions with their designation are provided in Table 3.

### 2.2. Methods

#### 2.2.1. Mixture Preparation and Consistency Verification

Due to the differing properties of the raw materials, two preparation methods were applied. For geopolymer composites, fly ash and slag were homogenised, followed by the addition of an alkaline solution, fibre-reinforced mortar, and finally foam, which was incorporated gradually to preserve pore integrity. The mixtures were cast and cured in moulds without mechanical compaction. For concrete with recycled additives, a dry fibre-reinforced mix and a stable foam of defined parameters were prepared separately and combined only after evaluation of foam density and homogeneity. Workability and stability were assessed, and process parameters were adjusted when necessary.

In both approaches, the objective was to obtain homogeneous, lightweight composites with properties suitable for 3D printing. Consistency was verified using a flow table test according to EN 1015-3 [47]. Detailed preparation procedures are reported in the authors’ previous articles [35,36,37].

#### 2.2.2. Printed Structures and Printing Process

**Figure 4:**
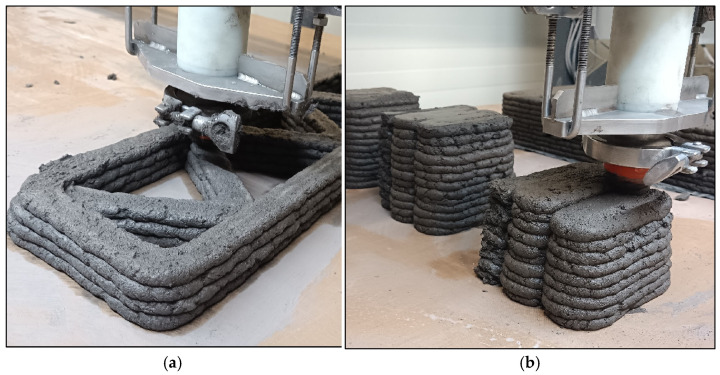
Printed elements for testing: (**a**) wall, (**b**) cube.

**Figure 5:**
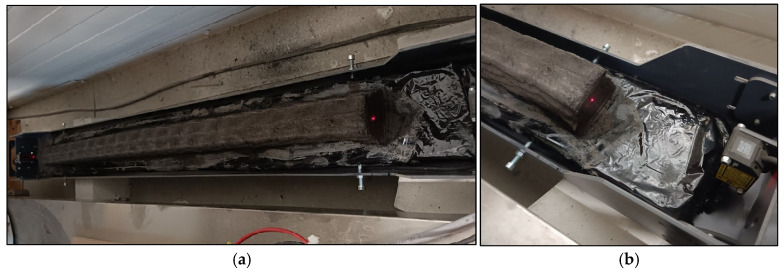
Experimental apparatus for measuring shrinkage in 3D-printed materials: (**a**) measurement from both sides of the specimen; (**b**) close-up view of the measurement system. The red laser spot represents the laser displacement sensor used for shrinkage measurements.

Printability tests were performed using a gantry-type 3D printer equipped with a 20 L hopper, a screw feeder, and a 20 mm nozzle. The material was deposited in layers with a height of 8 mm and a width of 20 mm at a printing speed of 40 mm/s. For each mixture, two printed wall segments (1.0 m in the X-direction and 0.5 m in the Z-direction), three cube specimens (150 × 150 × 150 mm), and two prismatic specimens (40 × 40 × 1000 mm) were manufactured under controlled laboratory conditions (22 ± 2 °C and 55 ± 5% relative humidity), as shown in Figure 4 and Figure 5. All mixtures exhibited sufficient printability to produce stable wall segments without visible collapse or excessive deformation during the printing process. One wall segment was kept under laboratory conditions as a reference, whereas the other was exposed to natural outdoor conditions for six months.

Following the exposure period, specimens (40 × 40 × 160 mm) were extracted from the printed elements and tested in flexure, compression, and shear. Cube specimens were designated for controlled freeze–thaw cycle testing, while prismatic specimens were used for shrinkage measurements.

#### 2.2.3. Shrinkage Test

Shrinkage behaviour was evaluated during the setting and early curing stages using prismatic specimens (40 × 40 × 1000 mm). 3DFCP specimens were placed on a low-friction support to allow for unrestricted deformation. Measurements were performed with a device equipped with two laser displacement sensors positioned at opposite ends of each specimen (Figure 5). A detailed description of the method can be found in the author’s previous publication [48].

#### 2.2.4. Freezing and Thawing Cycles

**Figure 6:**
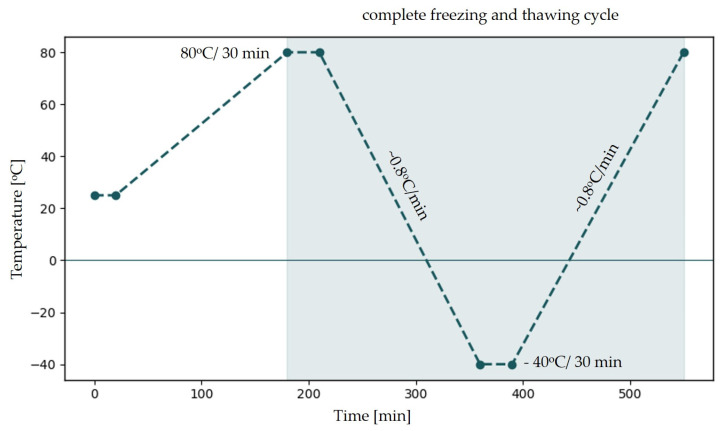
Freezing and thawing cycle. Temperature profile showing the initial conditioning cycle followed by successive freeze–thaw cycles (shaded area), including heating to 80 °C, cooling to −40 °C, and reheating at a rate of −0.8 °C/min.

Freeze–thaw cycles were carried out using a LabEvent LC/34/40/5 climatic chamber (WeissTechnik, Oldenburg, Germany). The experimental procedure involved subjecting the prepared mixtures to repeated temperature variations in the range of −40 °C to 80 °C, ensuring that the material repeatedly crossed the 0 °C threshold. A single cycle consisted of the following stages: cooling to −40 °C (150 min) and holding this temperature for 30 min, followed by heating to 80 °C (150 min) and maintaining this temperature for an additional 30 min. All stages were performed continuously in a cyclic mode. The duration of one complete cycle was 360 min. The total experimental period lasted 30 days, corresponding to 120 full cycles and 240 transitions through 0 °C (Figure 6).

**Figure 7:**
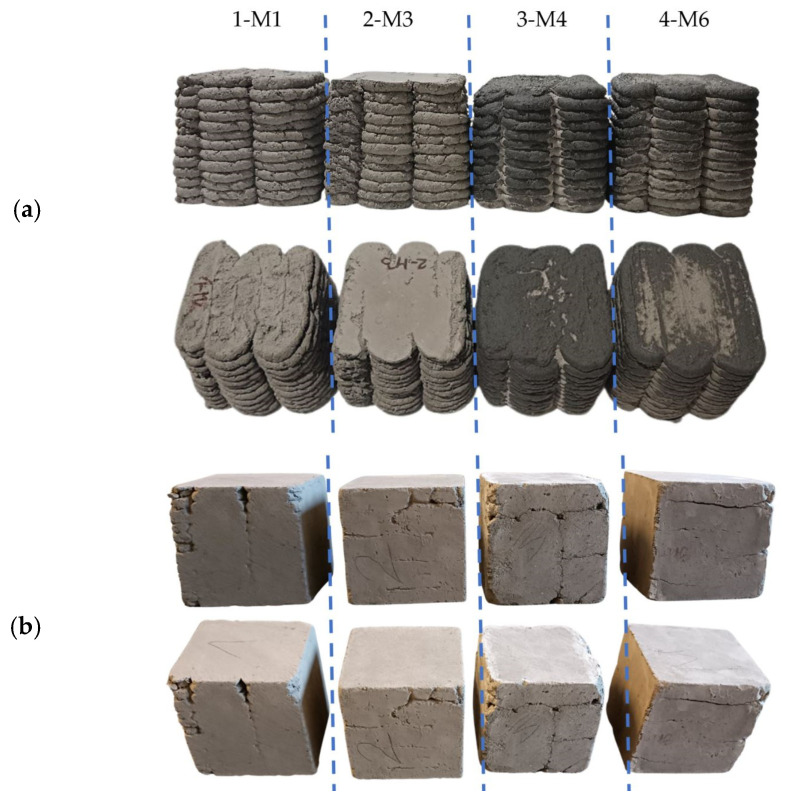
Layer morphology of 3D-printed samples: (**a**) lateral surface with distinct layer deposition, (**b**) the surface morphology of the cubic specimens.

As illustrated in Figure 7, the fabricated specimens displayed a wavy surface topology, with individual layers aligned orthogonally to the printing direction. To reduce the effect of surface unevenness on crack development, all specimens were mechanically smoothed prior to testing, since crack initiation most commonly occurs at the interfaces between successive layers. 

#### 2.2.5. Strength and Anisotropy Under Environmental Conditioning

**Figure 8:**
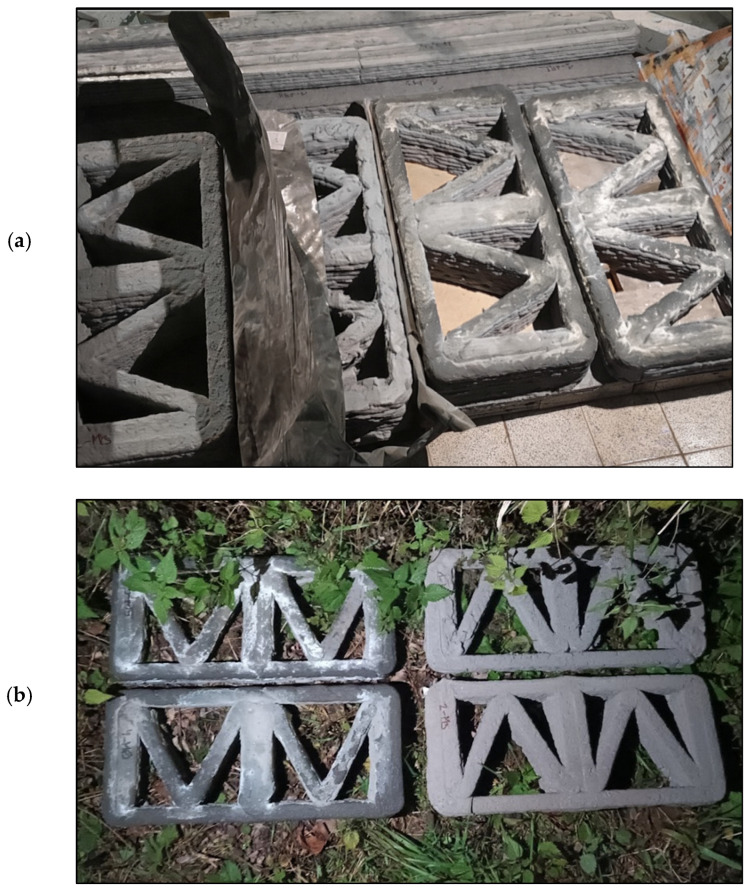
3D-printed wall elements: (**a**) specimens stored under laboratory conditions, (**b**) specimens exposed to natural atmospheric conditions for six months.

**Figure 9:**
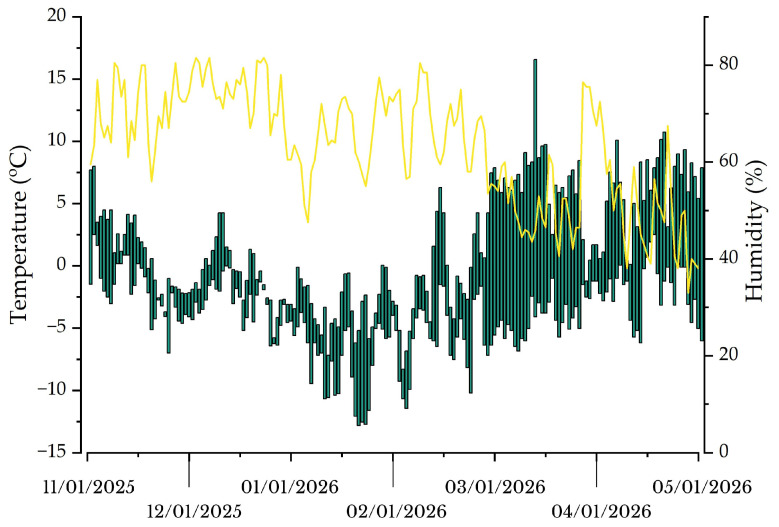
Meteorological parameters: air temperature and relative humidity over the investigated period. The green bars represent the daily air temperature (°C, left y-axis), while the yellow line represents the relative humidity (%, right y-axis).

**Figure 10:**
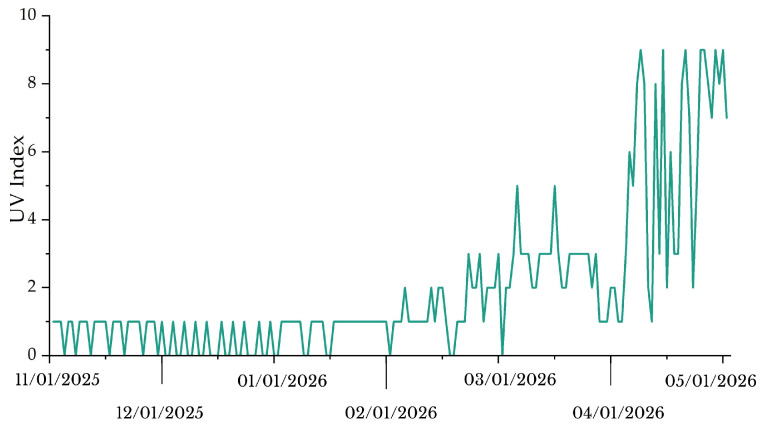
Changes in UV index values recorded throughout the measurement period.

To enable a comparative assessment of mixture performance after environmental exposure, two sets of 3D-printed walls were prepared. The first set was stored under stable laboratory conditions (22 °C ± 2 °C and 55% °C ± 3% humidity) and served as the reference specimens, while the second set was exposed to natural atmospheric conditions for a period of six months, from early November to late March, in a temperate climate zone (Figure 8). To characterise the environmental conditions during the exposure period, atmospheric parameters, including air temperature, relative humidity, and UV index, were continuously monitored using a local weather station (LSK_meteo, Kraków, Poland; 50.057° N, 20.041° E). The station was equipped with a VEVOR 7-in-1 wireless meteorological system (VEVOR, Shanghai, China). The collected climatic data were used to support the interpretation of changes in the mechanical performance of the 3D-printed concrete specimens subjected to natural weathering conditions (Figure 9 and Figure 10). After the exposure period, test specimens intended for mechanical strength testing were cut from the printed walls. Flexural strength was determined on 40 × 40 × 160 mm prisms according to PN-EN ISO 178 (three-point bending, 100 mm span, 5 mm/min) [49]. Compressive strength followed EN 12390-3 on 40 mm cubes at 1 mm/min [50]. Interlayer shear strength of 3D-printed specimens was measured under pure shear loading at 1 mm/min until interface failure, following the authors’ previous research. For each experimental variant, at least six independent analyses were performed to ensure the reproducibility of the results. All specimens, including the reference samples stored under laboratory conditions, the specimens exposed to natural outdoor conditions, and those subjected to freeze–thaw cycling, were mechanically tested after a total curing period of six months. Mechanical properties were evaluated using an MTS Criterion 43 universal testing system (MTS Systems Corp., Eden Prairie, MN, USA) with MTS TestSuites 1.0 software.

To assess the effect of environmental exposure on durability, compressive strength retention was determined by normalising the compressive strength measured after conditioning to the corresponding reference strength:

(1) $$Retention=\frac{f_{condition}}{f_{reference}}\times 100$$

where $f_{condition}$ is the compressive strength measured after atmospheric or freeze–thaw exposure, and $f_{reference}$ is the compressive strength of the corresponding reference specimens.

Strength retention was calculated separately for specimens loaded in the ⟂-direction, representing bulk material response, and in parallel (∥), to distinguish changes in matrix performance from changes related to interlayer bonding. A retention value of 100% indicates unchanged strength relative to the reference condition, values above 100% indicate strength gain, and values below 100% indicate deterioration after environmental exposure.

The degree of anisotropy $\left(I_{D}\right)$ was used to quantify the directional dependence of compressive strength resulting from the layered architecture of the printed composites. The adopted parameter is based on the anisotropy approach proposed in previous studies [51,52] for extrusion-based 3D-printed concrete. In the original formulation, the index was calculated using compressive strengths determined in three orthogonal directions [53]. Since only two loading directions were considered in the present study, the original approach was adapted by using the strengths measured parallel and perpendicular to the printing layers and their arithmetic mean as the reference value. This modification preserves the physical interpretation of the original index while providing a symmetric measure of anisotropy for the adopted experimental configuration. Following the concept proposed by [54], the anisotropy index was expressed as the normalised deviation of directional compressive strengths from their mean value.

First, the mean compressive strength was calculated according to Formula (2):

(2) $$\bar{f}=\frac{f_{⟂}+f_{∥}}{2}$$

The degree of anisotropy was then determined as:

(3) $$I_{D}=\frac{\sqrt{{(f}_{⟂}-\bar{f}{)}^{2}+{(f}_{∥}-\bar{f}{)}^{2}}}{\bar{f}}$$

where

$f_{∥}$ is the compressive strength measured in the direction sensitive to interlayer bonding,
and $f_{⟂}$ is the compressive strength measured in the perpendicular direction.

A value of $\left(I_{D}\right)$ = 0 indicates ideal isotropic behaviour, while increasing $\left(I_{D}\right)$ values indicate increasing anisotropy. Physically, the anisotropy index represents the normalised deviation in compressive strength between the investigated loading directions and therefore reflects the influence of the layered architecture and interlayer interfaces on the mechanical response of the printed material. Because the index is normalised by the mean compressive strength, it enables direct comparison of anisotropy between materials with different absolute strength levels. Changes in $\left(I_{D}\right)$ after environmental exposure were used to assess the evolution of interlayer-related anisotropic behaviour. In this way, the proposed index provides information not only on the degradation of compressive strength but also on whether environmental exposure affects both loading directions uniformly or preferentially.

#### 2.2.6. Microscopic Analysis

A detailed evaluation of the fibre structure and potential structural irregularities was performed based on microscopic observations conducted at various magnification levels. High-resolution images with an extended depth of field were acquired using a digital optical microscope (Keyence VHX-7000, KEYENCE International, Mechelen, Belgium) operating in multi-plane imaging mode. The applied methodology enabled a comprehensive characterisation of fibre morphology as well as the assessment of fibre degradation under different exposure conditions. Subsequent quantitative image analysis was carried out using Fiji ImageJ software (version IJ 1.52).

## 3. Results and Discussion

### 3.1. Mixture Properties and Printing Performance

**Figure 11:**
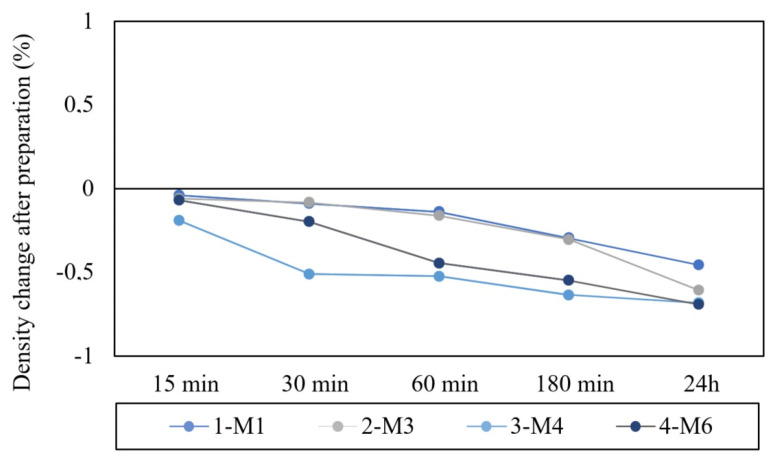
Percentage increase in the density of the tested mixtures over time.

The density of the investigated mixtures exhibited a slight decrease during the first 24 h after preparation (Figure 11). The observed variations remained below 0.7% for all formulations, indicating a high degree of stability of the foamed structure. These results suggest that the incorporated foam maintained its integrity throughout the monitoring period, with no evidence of significant pore collapse or structural degradation.

Among the tested formulations, mixture 1-M1 demonstrated the smallest density variation, indicating the highest stability of the foamed matrix. In contrast, mixture 3-M4 exhibited a slightly greater decrease in density, which may be associated with differences in the binder system rather than the fibre reinforcement. The alkali-activated matrix undergoes rapid physicochemical changes during the early stages after mixing, including ongoing geopolymerisation, redistribution of the liquid phase, and changes in the viscosity of the fresh mixture, which may slightly influence the stability of the foamed structure and consequently the measured density. Similar observations have been reported in previous studies [54,55,56], where minor reductions in fresh density were associated with physicochemical processes occurring within the foam immediately after mixing. In freshly prepared foamed systems, gradual drainage of the liquid phase from the inter-bubble films and Plateau borders may occur, accompanied by bubble coarsening resulting from gas diffusion between adjacent pores. These phenomena lead to slight modifications of the pore structure and can contribute to a modest decrease in the measured fresh density over time. Nevertheless, the limited magnitude of the density changes observed in the present study confirms the adequate stability of the foamed mixtures and the effectiveness of the adopted mix design in preserving the cellular structure during the early curing stage.

The flow diameter of the investigated mixtures gradually decreased with increasing time after preparation (Table 4), indicating a progressive loss of workability and the development of the internal structure within the alkali-activated matrix. The initial flow diameter varied between 136 and 154 mm and decreased to 102–120 mm after 30 min of resting time. This behaviour reflects the ongoing geopolymerisation reactions and water redistribution processes occurring within the fresh mixtures. Among the tested formulations, mixture 4-M6 exhibited the smallest reduction in flow diameter (25 mm), demonstrating the highest retention of rheological properties over time. In contrast, mixture 3-M4 showed the largest decrease in flowability (37 mm), indicating a more rapid structural build-up and loss of workability. The lower initial flow diameter observed for the alkali-activated mixtures compared with the cement-based mixtures is primarily associated with the rapid physicochemical reactions initiated immediately after mixing. The early dissolution of aluminosilicate precursors and the onset of geopolymerisation promote structural build-up and increase the viscosity of the fresh mixtures, resulting in reduced initial flowability [57,58]. Among the investigated alkali-activated mixtures, M4 exhibited a greater reduction in flow diameter than M6. Since the proportions of both glass fibres and merino wool fibres were modified simultaneously, the individual contribution of each fibre type cannot be unambiguously distinguished based on the present experimental programme. Therefore, the observed rheological differences are most likely associated with the combined influence of the hybrid fibre composition and its interaction with the alkali-activated matrix. Nevertheless, the lower workability generally reported for mixtures containing a higher proportion of natural fibres may be related to their hydrophilic nature, which promotes water absorption from the surrounding matrix and reduces the amount of free water available for lubrication between solid particles. Similar effects of natural fibre incorporation on rheological behaviour, water retention, structural build-up, and extrusion performance of cementitious and alkali-activated mixtures suitable for extrusion-based 3D printing have also been reported in previous studies [35,59].

### 3.2. Shrinkage Behaviour of the 3D-Printed Structures

**Figure 12:**
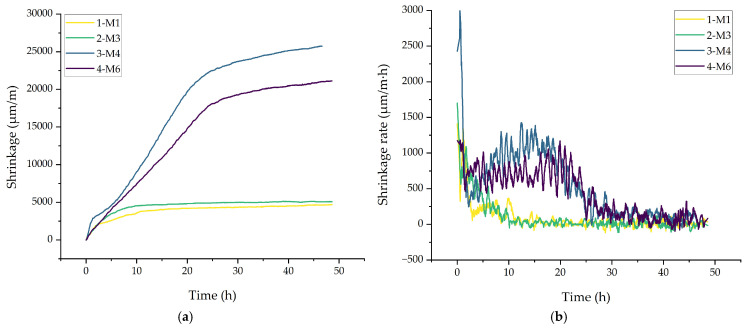
Effect of mixture composition on the time-dependent shrinkage behaviour of 3D-printed specimens; (**a**) shrinkage evolution over time, (**b**) shrinkage rate (time derivative of shrinkage), showing the rate of deformation over time.

The dimensional stability of the printed foamed composites was assessed by monitoring the evolution of shrinkage over time. The shrinkage changes depending on the composition of the investigated mixtures are presented in Figure 12. All tested mixtures exhibited a progressive increase in shrinkage with time. The highest rate of deformation was observed immediately after printing, indicating that the most significant dimensional changes occurred during the early period (Figure 12a). Subsequently, the rate of shrinkage gradually decreased, and the deformation curves approached a plateau, suggesting progressive stabilisation of the internal microstructure and a reduction in moisture-driven volumetric changes. Significant differences in shrinkage behaviour were observed between the cement-based and alkali-activated mixtures. The cement-based foamed composites (1-M1 and 2-M3) exhibited relatively low shrinkage values, reaching approximately 4500–5000 μm/m at the end of the monitoring period. In contrast, the alkali-activated foamed composites (3-M4 and 4-M6) developed substantially higher shrinkage, ranging from approximately 21,000 to 25,000 μm/m. This pronounced difference can be attributed to the distinct reaction mechanisms and microstructural evolution of alkali-activated binders. In alkali-activated slag systems, rapid gel formation, self-desiccation, and refinement of the pore structure frequently generate elevated capillary stresses, resulting in increased shrinkage compared with conventional cementitious materials [61,62].

The most intensive shrinkage development occurred during the first few hours after printing. This stage is associated with rapid moisture redistribution within the cellular pore network, evaporation of free water, and ongoing binder reaction processes. Simultaneously, progressive densification of the matrix and development of the solid skeleton contribute to volumetric contraction. As curing progresses and the microstructure becomes increasingly consolidated, the rate of shrinkage decreases, leading to stabilisation of the deformation response. The observed shrinkage behaviour during the initial curing period may contribute to the development of internal stresses, geometric deformations, and localised cracking, particularly in slender structural elements with high aspect ratios. Therefore, evaluating shrinkage characteristics is essential for assessing the suitability of such materials for 3D-printing applications and ensuring the dimensional accuracy of printed components [63]. Furthermore, the substantially higher shrinkage observed in alkali-activated mixtures compared to cement-based materials may pose an important limitation for large-scale 3D-printing applications in construction. Elevated early-age deformations can adversely affect dimensional accuracy, reduce buildability, and potentially weaken interlayer bonding due to shrinkage gradients between consecutively deposited layers [64].

Figure 12b shows the corresponding shrinkage rate, representing the time derivative of the shrinkage curves presented in Figure 12a. The shrinkage rate analysis confirms that the highest intensity of deformation occurs immediately after printing, followed by a continuous reduction in the rate as the material approaches a more stable state.

### 3.3. Effect of Environmental Exposure and Freeze–Thaw Cycles on Mechanical Performance

**Figure 13:**
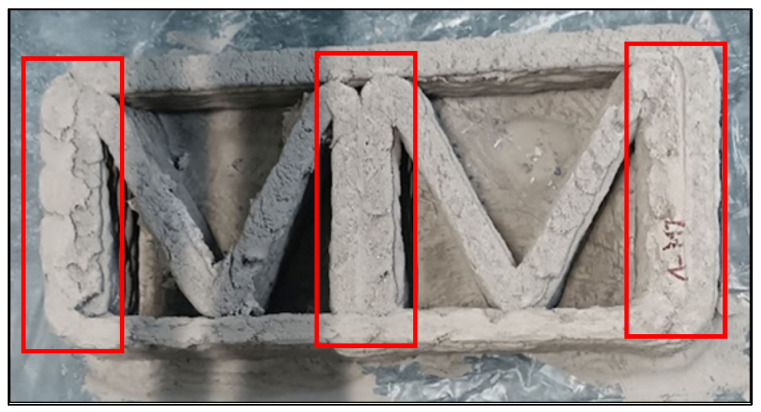
Representative view of specimen extraction locations in exterior and interior walls of 3DFCP elements for mechanical performance evaluation. The red rectangles indicate the regions cut from the walls for mechanical testing.

To assess the influence of environmental exposure and freeze–thaw cycles on the mechanical performance of the developed materials, 3DFCP specimens were collected from both exterior and interior wall sections of the printed elements. A representative example of the specimen extraction locations is presented in Figure 13. Such a sampling strategy allows for a more representative evaluation of the mechanical response of different regions within prospective structural components.

**Figure 14:**
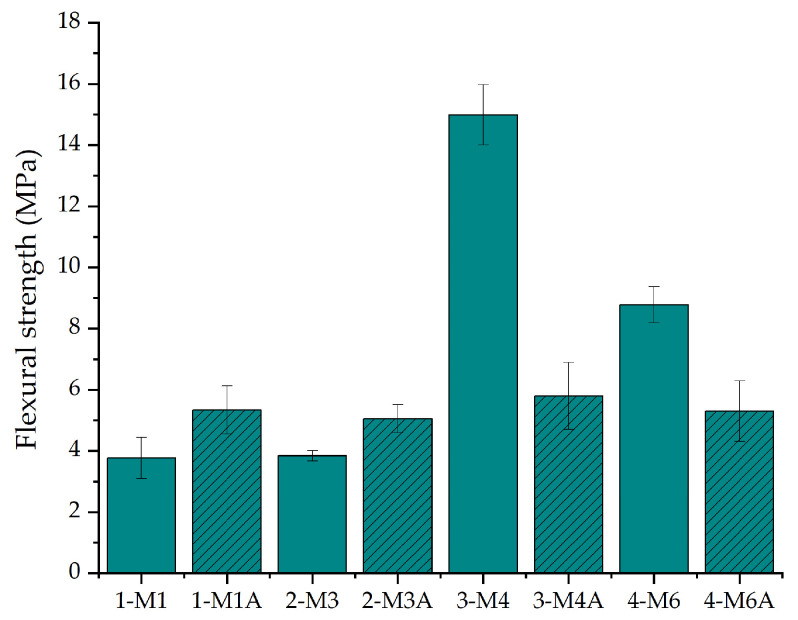
Effect of mixture composition and environmental exposure on the flexural strength of 3DFCP specimens. The letter “A” appended to the specimen designation indicates specimens tested after environmental exposure.

The flexural strength results presented in Figure 14 reveal distinct differences between the non-activated and alkali-activated mixtures. The non-activated mixtures exhibited relatively low flexural strength values with limited sensitivity to atmospheric exposure, ranging from 3.77 to 5.34 MPa for mixture 1-M1 and from 3.84 to 5.05 MPa for mixture 2-M3. The observed variations between the reference and exposed specimens remained relatively small, indicating a stable flexural response under the applied environmental conditions. In contrast, the alkali-activated mixtures demonstrated substantially higher flexural strength in the reference state, followed by a marked reduction after atmospheric exposure. For mixture 3-M4, the flexural strength decreased from 14.99 MPa to 5.80 MPa, corresponding to a reduction of approximately 61%. Similarly, mixture 4-M6 exhibited a decrease from 8.78 MPa to 5.30 MPa, representing a reduction of approximately 40%. These results indicate that although alkali activation significantly enhanced the initial flexural performance, the resulting material exhibited greater susceptibility to strength degradation under environmental exposure conditions. A similar relationship between increased initial mechanical performance of 3D-printed alkali-activated materials and their sensitivity to environmental action, particularly due to interlayer defects and anisotropic microstructure, has been reported in recent review papers on extrusion-based geopolymers and 3D-printed cementitious materials [65].

**Figure 15:**
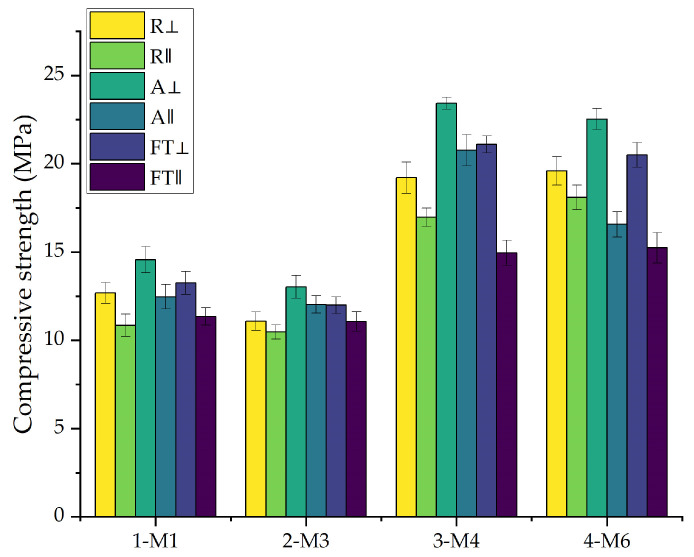
Effect of mixture composition and environmental conditioning on the compressive strength of 3DFCP specimens. The letter “R” in the specimen designation denotes reference conditions (standard laboratory environment), “A” indicates specimens subjected to atmospheric exposure, and “FT” refers to specimens conditioned under freeze–thaw cycling.

The compressive strength results presented in Figure 15 indicate a clear dependence on both mixture composition and environmental exposure conditions. The non-activated mixtures exhibited lower compressive strength values in the reference state, ranging from 10.85 to 12.68 MPa for mixture 1-M1 and from 10.47 to 11.08 MPa for mixture 2-M3. Following atmospheric exposure, a moderate increase in compressive strength was observed, reaching 12.03–14.57 MPa. The increase in compressive strength observed after long-term atmospheric exposure may be attributed to the continued hydration of the cementitious matrix and the ongoing geopolymerisation of the alkali-activated systems. These processes promote further microstructural densification and pore refinement, resulting in improved mechanical performance despite prolonged environmental exposure. Similar observations have been reported in previous studies on cementitious and alkali-activated materials exposed to long-term curing and environmental conditioning [66,67]. In contrast, freeze–thaw conditioning led to comparable or slightly reduced strength values, particularly in the parallel loading direction. The absence of strength deterioration in the perpendicular direction suggests that the bulk matrix remained largely unaffected by freeze–thaw cycling. This behaviour indicates that the investigated foamed composites possessed sufficient freeze–thaw resistance, while the detrimental effects of cyclic freezing and thawing were primarily concentrated within the weaker interlayer interfaces.

On the other hand, the alkali-activated mixtures demonstrated significantly higher compressive strength. For mixture 3-M4, the strength increased from 16.97–19.21 MPa in the reference condition to 20.77–23.43 MPa after atmospheric exposure. Similarly, mixture 4-M6 exhibited an increase from 18.09–19.60 MPa to 16.57–22.53 MPa, depending on the loading direction. After freeze–thaw exposure, the compressive strength remained at a comparable level in the perpendicular direction (21.09 MPa for 3-M4 and 20.50 MPa for 4-M6), whereas a reduction was observed in the interlayer direction (14.95 MPa for 3-M4 and 15.24 MPa for 4-M6), indicating pronounced anisotropy in mechanical response. When expressed relative to the reference specimens, the compressive strength in the perpendicular loading direction remained above 100% for all mixtures after both atmospheric exposure and freeze–thaw cycling, indicating that the bulk matrix was not adversely affected by environmental conditioning. In contrast, the alkali-activated mixtures exhibited a noticeable reduction in strength retention in the parallel loading direction after freeze–thaw exposure, confirming that environmental deterioration was concentrated primarily within the interlayer regions. This observation is consistent with previous studies showing that extrusion-based 3D printing inherently produces anisotropic mechanical properties due to weaker interlayer bonding and preferential orientation of deposited filaments, with the interlayer direction being the most vulnerable under mechanical and environmental loading [68,69].

**Figure 16:**
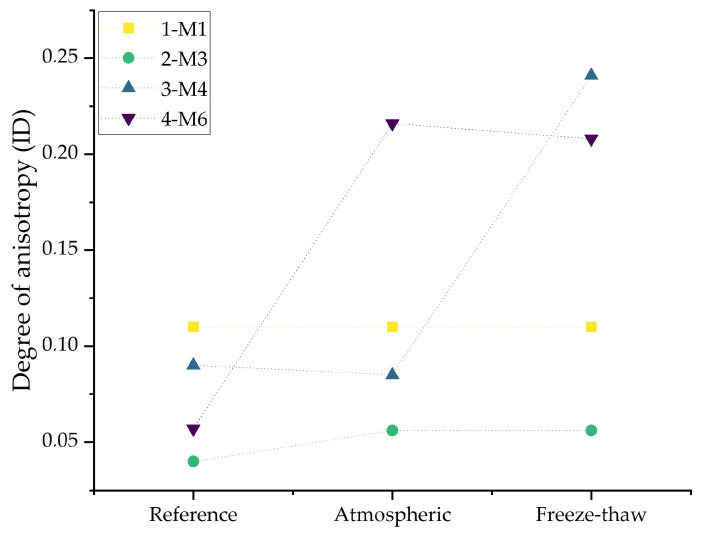
Evolution of anisotropy (*I_D_*) of 3DFCP composites as a function of mixture composition and environmental conditioning.

The evolution of the degree of anisotropy (*I_D_*) revealed pronounced differences in the environmental sensitivity of the investigated mixtures (Figure 16). The non-activated composites (1-M1 and 2-M3) exhibited relatively low and stable *I_D_* values after both atmospheric and freeze–thaw exposure, indicating preserved directional consistency and limited degradation of interlayer-related mechanical response.

In contrast, the alkali-activated mixtures showed distinctly different behaviour. Mixture 3-M4 remained largely stable after atmospheric exposure; however, a pronounced increase in *I_D_* was observed following freeze–thaw conditioning. Mixture 4-M6 exhibited a substantial increase in *I_D_* already after atmospheric exposure, which persisted and further stabilised under freeze–thaw cycling. These increases indicate an enhanced directional dependence of mechanical performance and suggest progressive degradation or weakening of interlayer bonding under environmental loading. Similar observations have been reported by To et al. [70], who demonstrated that the mechanical response of 3D-printed concrete is strongly governed by interlayer bond quality and that deterioration of these interfaces results in increasingly anisotropic compressive behaviour. Likewise, Heras Murcia et al. [71] reported significant directional dependence of compressive strength in 3D-printed concrete, with the lowest values obtained in the loading direction perpendicular to the deposited layers, highlighting the influence of the layered architecture on mechanical performance.

The results indicate that while alkali activation improved compressive performance in the perpendicular direction, it simultaneously increased the susceptibility of interlayer interfaces to environmentally induced anisotropic deterioration. This behaviour highlights a trade-off between mechanical strength enhancement and interfacial durability in 3D-printed cementitious composites. Recent reviews have similarly emphasised that the freeze–thaw durability of 3D-printed concrete is strongly governed by its anisotropic layered architecture and the quality of interlayer bonding, which make printed elements more vulnerable to environmental deterioration than conventionally cast concrete [72]. These findings are consistent with recent state-of-the-art reviews, which identify the quality of interlayer bonding as one of the key factors governing the anisotropic mechanical performance of extrusion-based 3D-printed concrete [73].

**Figure 17:**
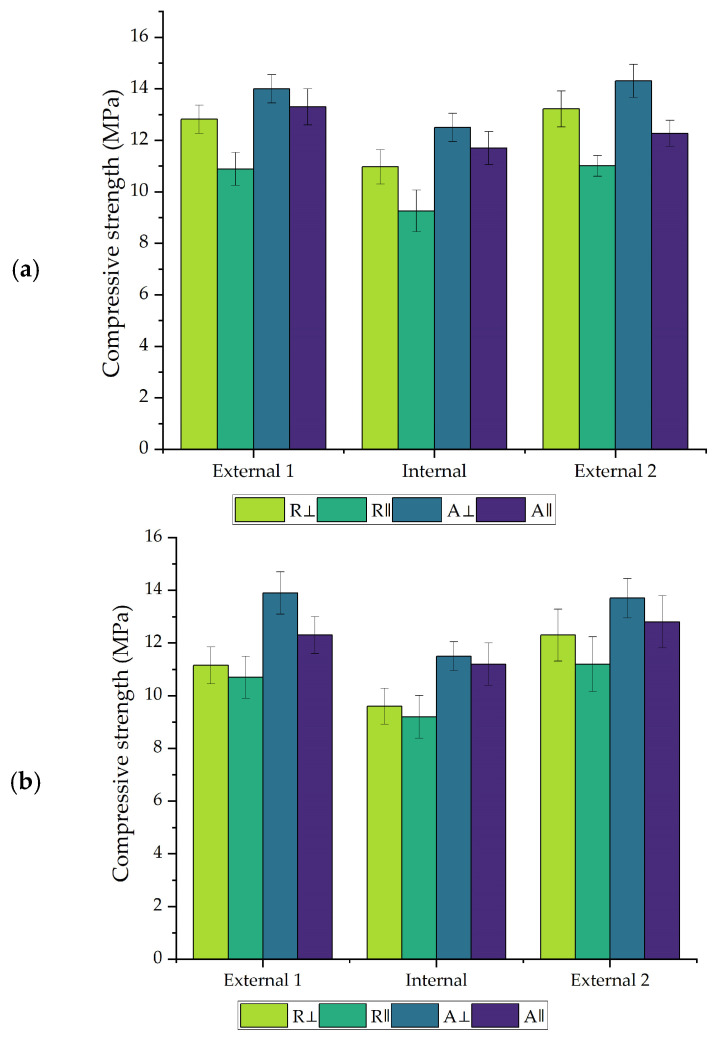

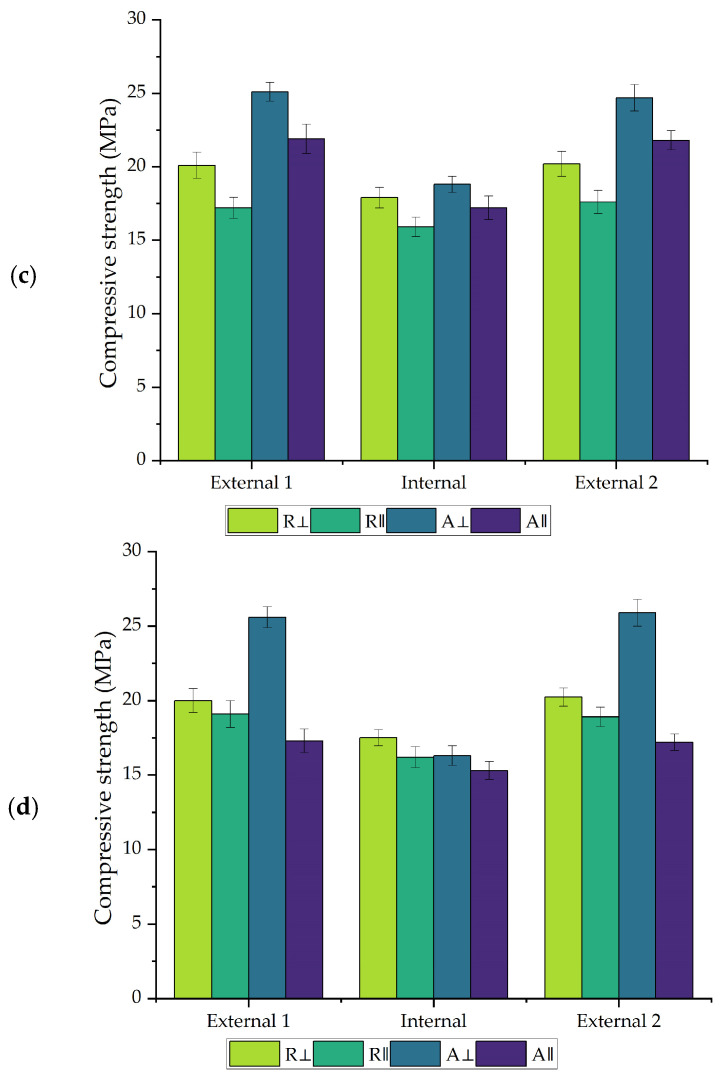
Compressive strength of wall sections of 3DFCP specimens under different loading directions and environmental conditioning as a function of mixture composition: (**a**) 1-M1, (**b**) 2-M3, (**c**) 3-M4, (**d**) 4-M6. The letter “R” in the specimen designation denotes reference conditions (standard laboratory environment), while “A” indicates specimens subjected to atmospheric exposure.

The results presented in Figure 17 indicate a consistent, although relatively small, tendency towards higher compressive strength in specimens extracted from the external wall regions compared with the internal region. While this observation may suggest some degree of spatial variability within the printed elements, the available experimental data are insufficient to identify the governing mechanism. Therefore, additional microstructural characterisation is required before definitive conclusions can be drawn.

### 3.4. Microstructural Interpretation

**Figure 18:**
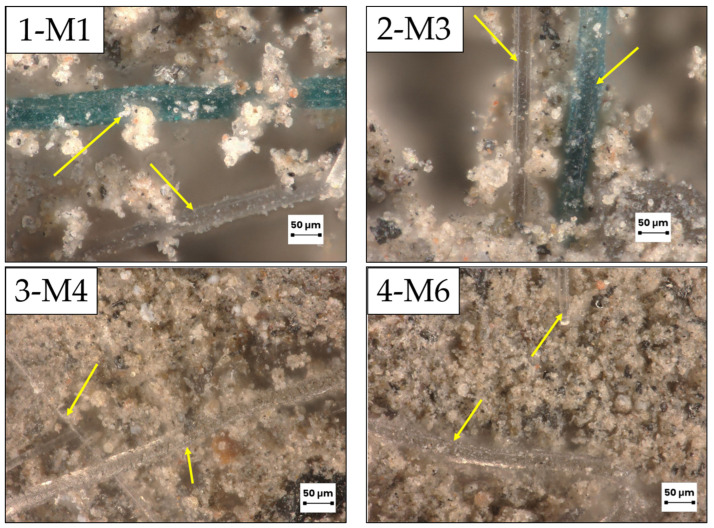
Representative view of 3DFCP composites, showing glass fibres (GFs) and merino wool fibres (MFs) cured under standard laboratory conditions. Arrows indicate the presence and orientation of the fibres. The rigid, transparent fibres correspond to glass fibres (GFs), whereas the flexible white and coloured fibres correspond to merino wool fibres (MFs).

**Figure 19:**
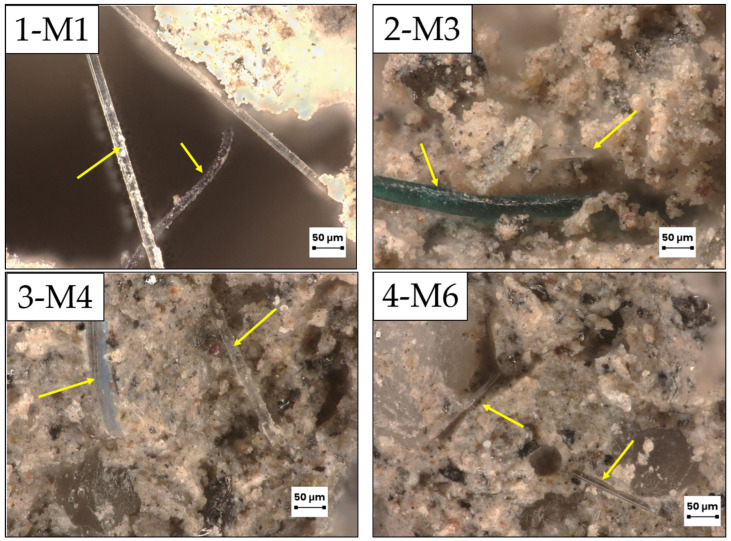
Representative views of 3DFCP composites, showing glass fibres (GFs) and merino wool fibres (MFs) after exposure in the environmental chamber. Arrows indicate the presence and orientation of the fibres. The rigid, transparent fibres correspond to glass fibres (GFs), whereas the flexible white and coloured fibres correspond to merino wool fibres (MFs).

**Figure 20:**
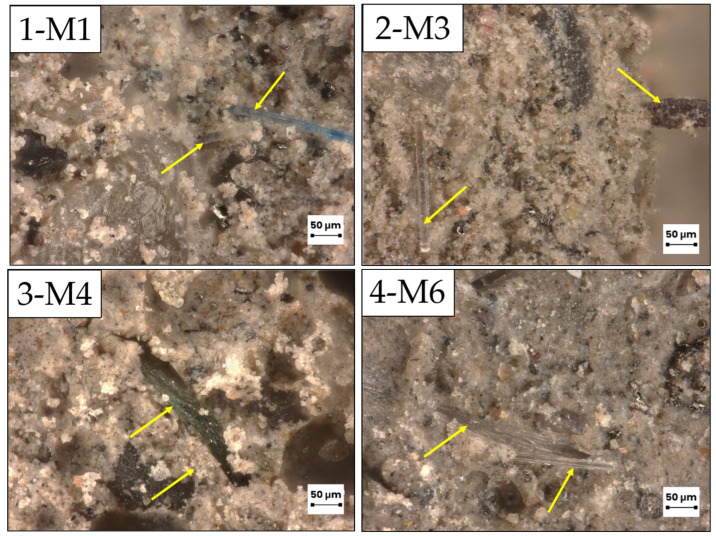
Representative view of 3DFCP composites, showing glass fibres (GFs) and merino wool fibres (MFs) stored under atmospheric conditions. Arrows indicate the presence and orientation of the fibres. The rigid, transparent fibres correspond to glass fibres (GFs), whereas the flexible white and coloured fibres correspond to merino wool fibres (MFs).

The microscopic images presented in Figure 18, Figure 19 and Figure 20 reveal distinct differences in the degradation mechanisms of glass fibres (GFs) and merino wool fibres (MFs) following environmental exposure and thermal cycling. The observed degradation behaviour is governed by both fibre morphology and the alkalinity, as well as the porosity of the cementitious/alkali-activated matrix. This behaviour is consistent with previous studies reporting that fibre durability in cementitious and geopolymer matrices is primarily controlled by matrix alkalinity, moisture transport, and the physicochemical stability of the fibre–matrix interface [74].

In the reference state, the glass fibres are characterised by a smooth and homogeneous surface without visible defects, which promotes efficient stress transfer and minimises stress concentrations at the fibre–matrix interface. In contrast, merino wool fibres exhibit a characteristic scaly surface morphology typical of keratin-based materials. This hierarchical structure increases the specific surface area but may also enhance moisture uptake and facilitate the ingress of alkaline species and hydration products into the fibre structure. Previous studies have shown that wool fibres may undergo limited chemical modification in alkaline environments through reactions involving keratin amino acids, while generally exhibiting better alkali resistance than many plant-derived fibres [72].

For the samples shown in Figure 19, no pronounced signs of advanced fibre degradation were observed for either fibre type, indicating overall stability under the initial exposure conditions. The degradation processes were predominantly superficial and were mainly associated with the deposition of hydration products and the onset of chemical interactions at the fibre–matrix interface. In sample 1-M1, the glass fibres retained their geometric continuity and smooth surface morphology; however, localised deposits of hydration products were detected on the fibre surface, suggesting initial stages of fibre–matrix interaction without evidence of cracking or reduction in the fibre cross-section. The merino wool fibres exhibited slight surface dulling accompanied by partial coverage with hydration products, while the characteristic scaly morphology remained clearly discernible, indicating limited keratin degradation at this stage.

In sample 2-M3, the glass fibres similarly maintained their structural integrity, whereas the merino wool fibres showed an increase in surface roughness and partial filling of interscale regions with matrix-derived products, indicating a progressive but still moderate advancement in interfacial interaction processes. The fibres tested in the activated samples (3-M4 and 4-M6) did not show significant signs of surface degradation. The merino wool fibres exhibited slightly raised surface scales and a more matte appearance compared to the as-received state; however, no structural damage, cracking, or fragmentation was observed. Overall, the fibres retained their integrity after exposure to the activated matrix, indicating good stability under the tested conditions.

As shown in Figure 20, for samples 1-M1 and 2-M3, both fibre types retained their overall geometric integrity after exposure in the climatic chamber. The merino wool fibres (MFs) did not exhibit visible fragmentation, swelling, or delamination of the characteristic scaly surface morphology. Similarly, the glass fibres (GFs) maintained a continuous structure without visible cracking or fibre separation. Only limited deposits of hydration products and fine mineral particles were observed on the fibre surfaces, indicating relatively weak physicochemical interactions between the fibres and the surrounding matrix. At the microscale, no clear evidence of severe degradation induced by freeze–thaw cycling or thermal stresses was identified.

On the other hand, for sample 3-M4, the first indications of surface degradation became apparent. The glass fibres lost their originally smooth morphology and exhibited localised surface dulling and micro-scale irregularities, which may suggest partial leaching of glass constituents under alkaline conditions. In the case of the merino wool fibres, a pronounced loss of surface regularity accompanied by localised constrictions was observed, indicating progressive degradation of the keratin-based structure.

For sample 4-M6, the glass fibres retained their original morphology and smooth surface after exposure in the climatic chamber. No visible signs of surface corrosion, fibre delamination, or significant structural deterioration were detected, confirming the good stability of glass fibres under combined freeze–thaw and thermal cycling conditions. In contrast, the merino wool fibres exhibited localised discontinuities and crack-like features. In one region, partial mechanical separation of the fibre was observed, which may indicate localised weakening of the keratin structure induced by cyclic environmental loading and moisture-related stresses. Despite these localised defects, no extensive surface degradation or complete disintegration of the wool fibres was observed.

After six months of exposure to atmospheric conditions, both glass fibres (GFs) and merino wool fibres (MFs) generally retained their structural integrity within the cementitious matrix. The observed changes were mainly limited to superficial mineral deposition and minor localised morphological irregularities, without clear evidence of severe fibre degradation. As shown in Figure 20, for sample 1-M1, the glass fibres maintained a continuous morphology with a smooth surface appearance, without visible cracking, delamination, or significant surface deterioration. Similarly, the adjacent merino wool fibres remained well embedded within the matrix and did not exhibit noticeable fragmentation or decomposition. Only localised mineral deposits and slight surface roughness were observed around the fibres, most likely associated with hydration products and prolonged environmental exposure. The merino wool fibres visible in the 2-M3 sample showed clearly opened (raised) surface scales; however, no signs of degradation or structural damage were observed. For sample 3-M4, the merino wool fibres exhibited minor surface irregularities and increased roughness; however, their overall geometry and continuity were preserved. No substantial degradation of the characteristic fibrous structure was identified. The observed changes may indicate limited physicochemical interaction between the wool fibres and the surrounding matrix during long-term exposure. The surrounding matrix showed partial deposition of fine mineral phases at the fibre interface, although without evidence of progressive deterioration.

The glass fibres of the 4-M6 sample, presented in Figure 20, retained their original morphology and remained largely resistant to atmospheric exposure. No clear signs of corrosion, cracking, or fibre disintegration were detected, confirming the high stability of glass fibres within the cementitious matrix. In contrast, the merino wool fibres exhibited localised surface irregularities and partial loosening of the fibrous structure. In some regions, slight fibrillation and reduced surface homogeneity were observed, suggesting a limited environmental effect on the keratin-based material. Nevertheless, the fibres generally preserved their continuity, and no severe degradation or complete structural breakdown was identified. The relatively limited degradation observed in the present study suggests that the investigated matrices provided sufficient protection against severe fibre deterioration during the applied exposure period, which is in agreement with previous reports indicating that geopolymer matrices may mitigate the degradation of natural fibres compared with conventional Portland cement systems [75]. Due to the highly porous and fragile nature of the investigated materials, reliable SEM/EDS specimen preparation was not feasible. Therefore, detailed characterisation of the fibre–matrix interface and degradation mechanisms using SEM/EDS is planned as part of future work.

## 4. Conclusions

This study demonstrates that the design and optimisation of 3D-printed cementitious and geopolymeric composites require a balanced consideration of fresh-state stability, shrinkage development, mechanical performance, and long-term interlayer durability. The investigated foamed mixtures exhibited satisfactory fresh-state stability, with density variations not exceeding 0.7% within the first 24 h, confirming a stable pore structure. However, all mixtures showed progressive loss of workability over time, while the alkali-activated systems became unsuitable for extrusion after approximately 30 min due to rapid structural build-up and reduced extrudability. Clear differences in shrinkage behaviour were observed between cement-based and alkali-activated systems. The cement-based composites exhibited relatively low shrinkage values (4500–5000 μm/m), whereas alkali-activated mixtures showed substantially higher shrinkage (21,000–25,000 μm/m), indicating increased susceptibility to early-age deformation and potential dimensional instability of printed elements. Alkali activation significantly enhanced the initial mechanical performance, particularly in terms of flexural and compressive strength, with the highest flexural strength recorded for mixture 3-M4 (14.99 MPa). However, environmental exposure and freeze–thaw cycling revealed a strong dependence of mechanical durability on mixture composition. While cement-based mixtures maintained relatively stable behaviour, alkali-activated composites exhibited pronounced reductions in flexural strength and interlayer performance degradation under freeze–thaw conditions. Despite this, compressive strength retention in the perpendicular (⟂) direction remained above 100% for all mixtures, indicating that bulk matrix performance was not adversely affected by environmental conditioning. In contrast, reduced strength retention in the parallel (∥) direction for alkali-activated mixtures confirmed increased anisotropy and progressive weakening of interlayer bonding. Finally, the results confirm that incorporating recycled materials such as fly ash, demolition brick powder, and coal slag enables the development of sustainable lightweight composites with promising mechanical performance and durability potential for extrusion-based additive manufacturing. Compared with previous studies on extrusion-based 3D-printed cementitious and geopolymer composites, which have primarily focused on conventional single-fibre reinforcement and short-term mechanical performance, the present study broadens the current state of knowledge by investigating hybrid reinforcement composed of two waste-derived fibres (glass fibres and waste merino wool fibres) incorporated into lightweight foamed cementitious and alkali-activated composites. The findings demonstrate that combining glass fibres with waste merino wool fibres represents a viable hybrid reinforcement strategy for lightweight 3D-printed composites, contributing to both improved mechanical performance and the circular utilisation of industrial and textile waste. Moreover, unlike most previous investigations, the durability of the printed composites was evaluated after both long-term natural outdoor exposure and freeze–thaw cycling while considering the influence of printing-induced anisotropy. The results further confirm that environmental degradation was concentrated primarily within the interlayer regions rather than the bulk matrix, highlighting the critical role of interlayer bonding in the long-term durability of extrusion-based printed materials. These results may suggest that the outer wall region possesses a denser and more homogeneous microstructure than the inner wall region; however, this hypothesis requires direct confirmation by pore structure characterisation techniques, such as mercury intrusion porosimetry, in future studies. Future research will focus on long-term durability studies of large-scale 3D-printed prefabricated elements exposed to natural environmental conditions, providing essential knowledge for the practical implementation of 3D concrete printing in the construction industry. In addition, further investigations will address the spatial distribution and orientation of fibres within the printed material, their influence on mechanical anisotropy, and the environmental and human health impacts of the developed composite to support its continued optimisation and sustainable application.

## Figures and Tables

**Table 1:** Morphological characteristics of the materials used for the preparation of the specimen matrix.

Material	Typical Particle Shape	Aspect Ratio of Fibre Length (L) to Fibre Diameter (D) (L/D)	Flakiness Index (FI, %)	Notes	
Cement	Irregular, mostly isometric	~1.1–1.5	<5%	Product of grinding, little tendency to form flat particles	[38,39]
FA	Spherical (cenospheres)	≈1.0	≈0%	Very high sphericity, often circularity > 0.9	[40,41]
SL	Irregular, often angular–platy	~1.5–2.5	10–25%	Product of rapid cooling and grinding, heterogeneous particle shapes	[42,43]
GB	Angular, platy fragments	~2–3	20–40%	Brittle ceramic material, dominated by flat and elongated particles	[44,45]

**Table 2:** Fibre characteristics.

Fibres	Aspect Ratio (L/D)	Humidity [%]
Glass (GFs)	300.00	12.7
Merino wool (MFs)	333.33	9.2

**Table 3:** Composition of mixtures and their designation.

Activated	Mixtures	w/s	Cement [wt.%]	Sand [wt.%]	FA [wt.%]	SL [wt.%]	GB [wt.%]	GF [wt.%]	MF [wt.%]
No	1-M1	0.34	27	37	5	15	15	0.3	0.2
No	2-M3	0.34	27	37	5	15	15	0.25	0.25
Yes	3-M4	0.34	27	37	5	15	15	0.3	0.2
Yes	4-M6	0.34	27	37	5	15	15	0.25	0.25

**Table 4:** Flow diameter of the mixtures measured at different times after preparation, according to PN-EN 1015-3 [60].

	Time from Preparation (min)		
	1	15	30
	Flow Diameter (mm)		
1-M1	154	137	120
2-M3	149	130	119
3-M4	139	123	102
4-M6	136	125	111

## Data Availability

The original contributions presented in this study are included in the article. Further inquiries can be directed to the corresponding author.
